# Establishment and Epitope Mapping of Anti-Diacylglycerol Kinase α Monoclonal Antibody DaMab-8 for Immunohistochemical Analyses

**DOI:** 10.1089/mab.2019.0004

**Published:** 2020-02-14

**Authors:** Masato Sano, Mika K. Kaneko, Hiroyoshi Suzuki, Yukinari Kato

**Affiliations:** ^1^ Department of Antibody Drug Development, Tohoku University Graduate School of Medicine, Sendai, Japan.; ^2^ Department of Pathology and Laboratory Medicine, Sendai Medical Center, Sendai, Japan.; ^3^ New Industry Creation Hatchery Center, Tohoku University, Sendai, Japan.

**Keywords:** diacylglycerol kinase, DGKα, monoclonal antibody, epitope mapping

## Abstract

Diacylglycerol kinase (DGK) converts diacylglycerol (DG) into phosphatidic acid (PA). DGKα, 1 of the 10 DGK isozymes, is involved in T cell function. In the present study, we describe a specific monoclonal antibody DaMab-8 (mouse IgG_1_, kappa) against DGKα, which is extremely useful for performing immunohistochemical analysis for T cells in oropharyngeal squamous cell carcinomas. Furthermore, we characterized the binding epitope of DaMab-8 using Western blotting and found that the sites Asn610, Leu611, Trp612, Gly613, Asp614, His619, Tyr623, and Gly624 of DGKα are important for facilitating the DaMab-8 binding to the DGKα protein. Thus, DaMab-8 could be advantageous for immunohistochemical analyses toward clarifying the distribution of DGKα-expressing T cells in every pathophysiological tissue.

## Introduction

Diacylglycerol kinase (DGK) phosphorylates diacylglycerol (DG) to produce phosphatidic acid (PA).^(1,2)^ DG is a neutral lipid derived from various sources, including phosphatidylinositol 4,5-bisphosphate and phosphatidylcholine, and it serves as a second messenger that activates the conventional and novel types of the protein kinase C (PKC) family, RasGRP, Unc-13, and canonical transient receptor potential channels.^(2,3)^ PA functions as a messenger molecule that activates the hypoxia-inducible factor (HIF)-1α, atypical PKCζ, and mammalian target of rapamycin. DGK constitutes an enzyme family comprising 10 isozymes of the mammalian species.^(1,2)^ Each isozyme possesses a distinct molecular structure and a subcellular localization pattern. DGKα is the first identified enzyme of 80-kDa size that contains an EF-hand motif (Ca^2+^-binding site), a Zn finger (C1 domain, DG-binding site), and a catalytic domain. DGKα regulates cell proliferation in response to IL-2 stimulation in T cells^(3)^ and is involved in T cell receptor (TCR) signaling via the modulation of the RasGRP activity.^(4)^ T cells isolated from DGKα-deficient mice demonstrate an altered activity of TCR signaling and hyperproliferation.^(5)^ DGKα is expressed in T lymphocytes abundantly, in which it facilitates the nonresponsive state known as clonal anergy.^(5)^ Anergy induction in T cells represents the main mechanism by which advanced tumors avoid immune action.^(6)^

Because only few specific anti-DGKα monoclonal antibodies (mAbs) are available to detect human DGKα using immunohistochemistry, the localization of DGKα-expressing cells remains unclear. Recently, we have developed DaMab-2 (mouse IgG_1_, kappa), a specific mAb against DGKα.^(7)^ DaMab-2 is extremely useful in immunocytochemical analysis using HeLa cells. We further characterized the binding epitope of DaMab-2 using Western blotting and revealed that the Cys246, Lys249, Pro252, and Cys253 sites of DGKα are important for facilitating DaMab-2 binding to the DGKα protein.^(8)^ However, DaMab-2 was not applicable for immunohistochemical analysis.

In the present study, we report a novel anti-human DGKα mAb DaMab-8 (mouse IgG_1,_ kappa) that is extremely useful in immunohistochemical analysis. Furthermore, we have characterized the binding epitope of DaMab-8 using Western blotting.

## Materials and Methods

### Plasmid preparation

Human DGKα cDNA^(9)^ was synthesized and subcloned into the expression vector pMAL-c2 (New England Biolabs, Inc., Beverly, MA), along with PA tag (GVAMPGAEDDVV),^(10)^ using the In-Fusion HD Cloning Kit (Takara Bio, Inc., Shiga, Japan); the resultant construct was named pMAL-c2-DGKα-PA. The deletion mutants of DGKα produced using PCR were subcloned into pMAL-c2 with PA tag using the In-Fusion PCR Cloning Kit. The substitution of DGKα amino acids 605–630 with either alanine or glycine in dN561 of DGKα was performed using the QuikChange Lightning Site-Directed Mutagenesis Kit (Agilent Technologies, Inc., Santa Clara, CA). These constructs were verified using direct DNA sequencing.

### Western blotting

Competent *Escherichia coli* TOP-10 cells (Thermo Fisher Scientific, Inc., Waltham, MA) were transformed and cultured overnight at 37°C in Luria–Bertani medium (Thermo Fisher Scientific, Inc.,) containing 100 μg/mL ampicillin (FUJIFILM Wako Pure Chemical Corporation, Osaka, Japan). The cell pellets were resuspended in phosphate-buffered solution containing 1% Triton X-100 and 50 μg/mL aprotinin (Sigma-Aldrich). Lysates were immunoprecipitated using amylose resin (New England Biolabs, Inc.) and boiled in sodium dodecyl sulfate (SDS) sample buffer (Nacalai Tesque, Inc., Kyoto, Japan). The samples were electrophoresed on 5%–20% polyacrylamide gels (FUJIFILM Wako Pure Chemical Corporation) and transferred onto a polyvinylidene difluoride membrane (Merck KGaA, Darmstadt, Germany). After blocking with 4% skim milk (Nacalai Tesque, Inc.) for 1 hour, the membrane was incubated with DaMab-8 for 1 hour, followed by peroxidase-conjugated anti-mouse IgG (1:2000 dilution; Agilent Technologies, Inc.) for 1 hour. The membrane was also incubated with NZ-1 (anti-PA tag) for 1 hour, followed by biotin-conjugated anti-rat IgG (1:1000 dilution; Agilent Technologies, Inc.) for 30 minutes, and further incubated with the avidin–biotin complex (Vector laboratories, Inc., Burlingame, CA) for 30 minutes. The membrane was finally developed with the ImmunoStar LD Chemiluminescence Reagent (FUJIFILM Wako Pure Chemical Corporation) using the Sayaca-Imager (DRC Co., Ltd., Tokyo, Japan). All procedures of Western blotting were performed at room temperature.

### Immunohistochemical analyses

Our study examined a patient with oropharyngeal squamous cell carcinoma who underwent surgery at the Sendai Medical Center. Informed consent for sample procurement and subsequent data analyses was obtained from the patient or the patient's guardian. The tissue samples were processed to produce 4-μm paraffin-embedded tissue sections that were directly autoclaved in citrate buffer (pH 6.0; Nichirei Biosciences, Inc., Tokyo, Japan) for 20 minutes and blocked using the SuperBlock T20 (PBS) Blocking Buffer (Thermo Fisher Scientific, Inc.,), incubated with DaMab-8 (1 μg/mL) for 1 hour at the room temperature, and then treated using the Envision Kit (Agilent Technologies, Inc.) for 30 minutes. The tissue sections were stained using 3,3′-diaminobenzidine tetrahydrochloride (DAB; Agilent Technologies, Inc.) for 2 minutes, and counterstaining was performed using hematoxylin (FUJIFILM Wako Pure Chemical Corporation).

## Results and Discussion

**FIG. 1.**
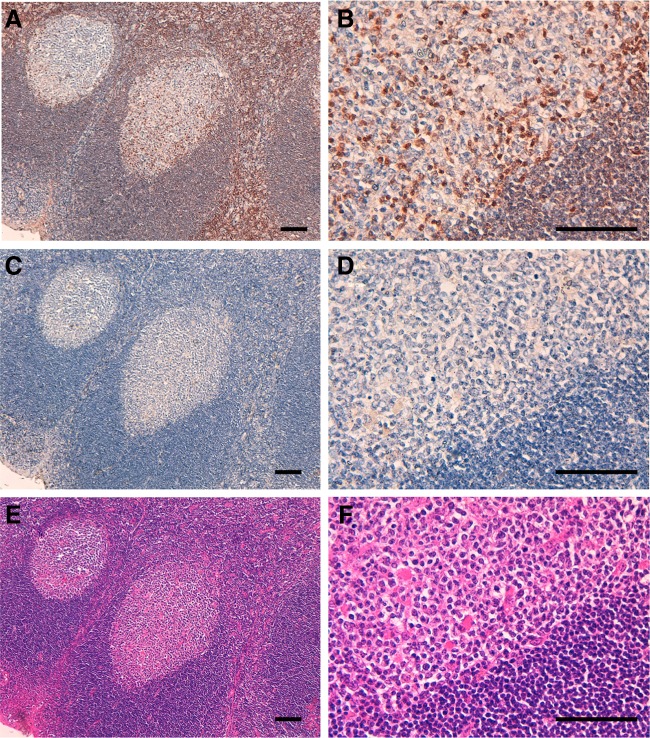
Immunohistochemical analysis using DaMab-8 against oropharyngeal squamous cell carcinomas. Tissue sections were incubated with DaMab-8 **(**1 μg/mL; **A, B)** or blocking buffer **(C, D)** for 1 hour at room temperature and treated using the Envision Kit (Agilent Technologies, Inc.) for 30 minutes. Scale bar = 100 μm. **(E, F)** Hematoxylin and eosin staining.

Several anti-DGKα mAbs are commercially available and are reportedly useful in Western blotting and for immunohistochemical analyses.^(11)^ Furthermore, we have developed DaMab-2 (mouse IgG_1_, kappa), a specific mAb against DGKα, which is extremely useful in immunocytochemical analysis.^(7)^ The binding epitopes of DaMab-2 were determined to be Cys246, Lys249, Pro252, and Cys253 of DGKα.^(8)^ Unfortunately, DaMab-2 was not applicable for immunohistochemical analysis. We have previously immunized mice with recombinant DGKα and developed several anti-DGK clones.^(7)^ One of these clones, DaMab-8, recognized only DGKα in ELISA and showed no reaction with other isozymes, such as DGKγ, DGKζ, DGKη, and DGKδ (data not shown). DGKα was reported to be expressed in T lymphocytes abundantly, in which it facilitates the nonresponsive state known as clonal anergy.^(5)^ Immunohistochemical screening revealed that DaMab-8 was extremely useful in immunohistochemical analysis for T cells in oropharyngeal squamous cell carcinomas (Fig. 1).

**FIG. 2.**
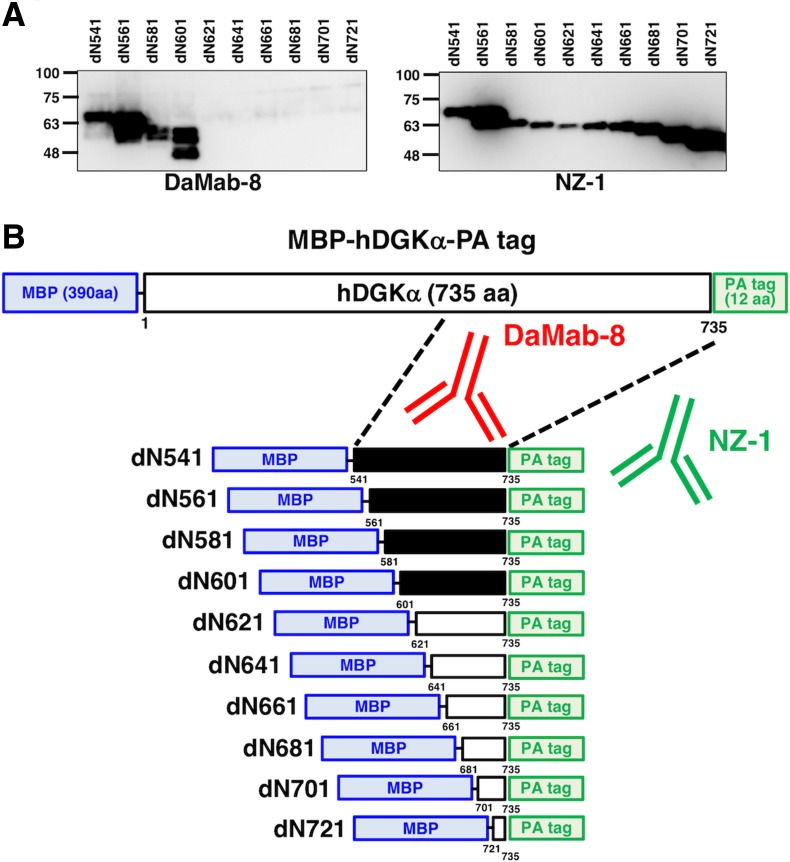
Production of DGKα deletion mutants. **(A)** Immunoprecipitates of deletion mutants were electrophoresed and transferred onto PVDF membranes. After blocking, the membranes were incubated with 1 μg/mL of DaMab-8 or anti-PA tag (NZ-1), followed by incubation with peroxidase-conjugated anti-mouse or anti-rat IgG. **(B)** Schematic illustration of DaMab-8 epitope. Black bars, deletion mutants detected by DaMab-8; white bars, deletion mutants not detected by DaMab-8. hDGKα, human diacylglycerol kinase α; MBP, maltose-binding protein; PVDF, polyvinylidene difluoride.

**FIG. 3.**
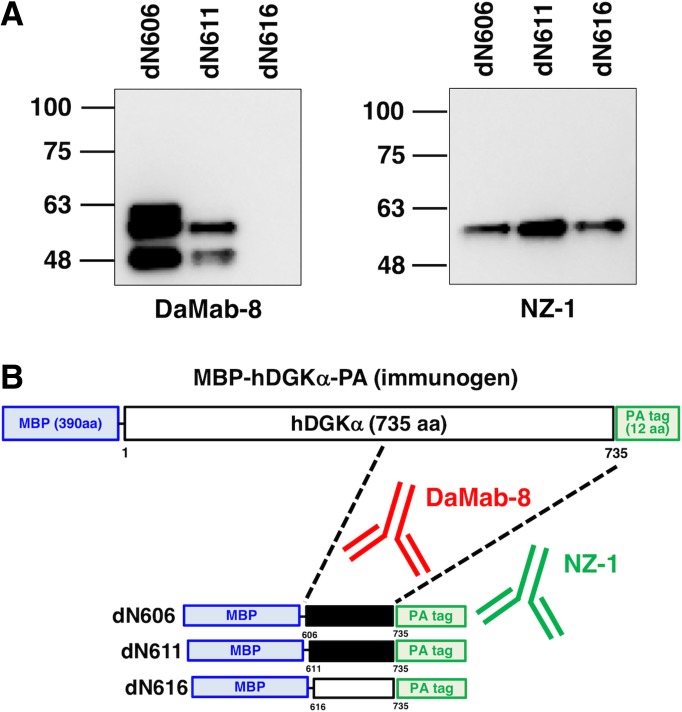
Production of additional deletion mutants of DGKα. **(A)** Immunoprecipitates of deletion mutants were electrophoresed and transferred onto PVDF membranes. After blocking, the membranes were incubated with 1 μg/mL of DaMab-8 and anti-PA tag (NZ-1), followed by incubation with peroxidase-conjugated anti-mouse IgG and biotin-conjugated anti-rat IgG, respectively. **(B)** Schematic illustration of DaMab-8 epitope. Black bars, deletion mutants detected by DaMab-8; white bars, deletion mutants not detected by DaMab-8.

We next moved on to the determination of the binding epitope of DaMab-8. As shown in Figure 2, we produced 10 N-terminal deletion mutants of DGKα (i.e., dN541, dN561, dN581, dN601, dN621, dN641, dN661, dN681, dN701, and dN721). Western blotting demonstrated that DaMab-8 detected dN541, dN561, dN581, and dN601 but not dN621, dN641, dN661, dN681, dN701, and dN721, although all deletion mutants were detected by an anti-PA tag mAb, NZ-1 (Fig. 2A), indicating that the N-terminus of the DaMab-8-epitope exists between amino acids 601 and 621 (Fig. 2B). Western blotting of the additional deletion mutants of DGKα—that is, dN606, dN611, and dN616—demonstrated that DaMab-8 detected dN606 and dN611, but not dN616, although all deletion mutants were detected by an anti-PA tag mAb, NZ-1 (Fig. 3A), thereby indicating that the N-terminus of the DaMab-8-epitope exists between amino acids 611 and 616 (Fig. 3B).

**FIG. 4.**
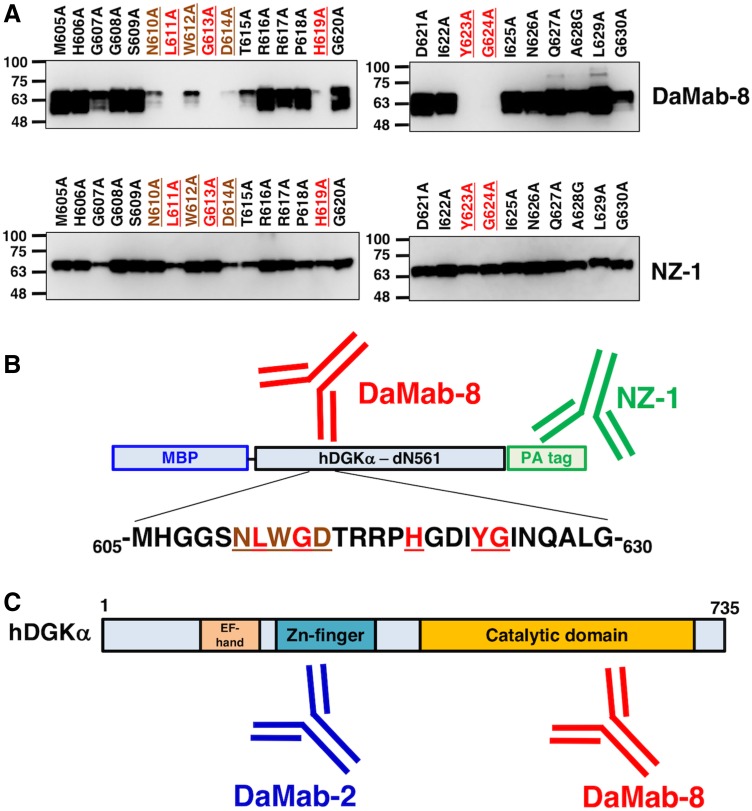
Epitope mapping of DaMab-8 using point mutants of DGKα. **(A)** Immunoprecipitates of point mutants were electrophoresed and transferred onto PVDF membranes. After blocking, the membranes were incubated with 1 μg/mL of DaMab-8 and anti-PA tag (NZ-1), followed by incubation with peroxidase-conjugated anti-mouse IgG and biotin-conjugated anti-rat IgG, respectively. **(B)** Schematic illustration of DaMab-8 epitope. **(C)** Schematic illustration of DaMab-2 and DaMab-8 epitopes. Red amino acids, strong reaction with DaMab-8; brown amino acids, weak reaction with DaMab-8. PVDF, polyvinylidene difluoride.

Accordingly, we produced the following 26 DGKα point mutants: M605A, H606A, G607A, G608A, S609A, N610A, L611A, W612A, G613A, D614A, T615A, R616A, R617A, P618A, H619A, G620A, D621A, I622A, Y623A, G624A, I625A, N626A, Q627A, A628G, L629A, and G630A. Western blotting demonstrated that the anti-PA tag mAb, NZ-1, detected all point mutants (Fig. 4A). In contrast, DaMab-8 strongly detected M605A, H606A, G607A, G608A, S609A, T615A, R616A, R617A, P618A, G620A, D621A, I622A, I625A, N626A, Q627A, A628G, L629A, and G630A; weakly detected N610A, W612A, and D614A; and did not detect mutants L611A, G613A, H619A, Y623A, and G624A (Fig. 4A). DaMab-8 epitopes are summarized in Figure 4B.

In conclusion, Asn610, Leu611, Trp612, Gly613, Asp614, His619, Tyr623, and Gly624 are important for facilitating DaMab-8 binding to the DGKα protein. This epitope exists in the catalytic domain of DGKα, whereas DaMab-2 binds to Zn-finger (Fig. 4C). DaMab-8 could be advantageous for immunohistochemical analyses toward clarifying the distribution of DGKα-expressing T cells in every pathophysiological tissue. Furthermore, these findings could be applied for the production of more functional anti-DGKα mAbs.

## References

[1] Topham MK, and Epand RM: Mammalian diacylglycerol kinases: molecular interactions and biological functions of selected isoforms. Biochim Biophys Acta 2009;1790:416–424 19364481 10.1016/j.bbagen.2009.01.010 PMC2744455

[2] Goto K, Hozumi Y, Nakano T, Saino SS, and Kondo H: Cell biology and pathophysiology of the diacylglycerol kinase family: morphological aspects in tissues and organs. Int Rev Cytol 2007;264:25–63 17964921 10.1016/S0074-7696(07)64002-9

[3] Sakane F, Imai S, Kai M, Yasuda S, and Kanoh H: Diacylglycerol kinases: why so many of them?. Biochim Biophys Acta 2007;1771:793–806 17512245 10.1016/j.bbalip.2007.04.006

[4] Jones DR, D'Santos CS, Merida I, and Divecha N: T lymphocyte nuclear diacylglycerol is derived from both de novo synthesis and phosphoinositide hydrolysis. Int J Biochem Cell Biol 2002;34:158–168 11809418 10.1016/s1357-2725(01)00108-x

[5] Olenchock BA, Guo R, Carpenter JH, Jordan M, Topham MK, Koretzky GA, and Zhong XP: Disruption of diacylglycerol metabolism impairs the induction of T cell anergy. Nat Immunol 2006;7:1174–1181 17028587 10.1038/ni1400

[6] Foell J, Hewes B, and Mittler RS: T cell costimulatory and inhibitory receptors as therapeutic targets for inducing anti-tumor immunity. Curr Cancer Drug Targets 2007;7:55–70 17305478 10.2174/156800907780006841

[7] Nakano T, Ogasawara S, Tanaka T, Hozumi Y, Mizuno S, Satoh E, Sakane F, Okada N, Taketomi A, Honma R, Nakamura T, Saidoh N, Yanaka M, Itai S, Handa S, Chang YW, Yamada S, Kaneko MK, Kato Y, and Goto K: DaMab-2: anti-human DGKalpha monoclonal antibody for immunocytochemistry. Monoclon Antib Immunodiagn Immunother 2017;36:181–184 28742439 10.1089/mab.2017.0023

[8] Sano M, Kaneko MK, and Kato Y: Epitope mapping of antidiacylglycerol kinase alpha monoclonal antibody DaMab-2. Monoclon Antib Immunodiagn Immunother 2019;38:8–11 30648914 10.1089/mab.2018.0047

[9] Schaap D, de Widt J, van der Wal J, Vandekerckhove J, van Damme J, Gussow D, Ploegh HL, van Blitterswijk WJ, and van der Bend RL: Purification, cDNA-cloning and expression of human diacylglycerol kinase. FEBS Lett 1990;275:151–158 2175712 10.1016/0014-5793(90)81461-v

[10] Fujii Y, Kaneko M, Neyazaki M, Nogi T, Kato Y, and Takagi J: PA tag: a versatile protein tagging system using a super high affinity antibody against a dodecapeptide derived from human podoplanin. Protein Expr Purif 2014;95:240–247 24480187 10.1016/j.pep.2014.01.009

[11] Wang Y, Zhang Q, Ma Q, Zhang Y, Li Z, and Wang C: DGKalpha DNA vaccine relieves airway allergic inflammation in asthma model possibly via induction of T cell anergy. Int J Clin Exp Pathol 2013;6:2404–2411 24228102 PMC3816809

